# MyoGestic: EMG interfacing framework for decoding multiple spared motor dimensions in individuals with neural lesions

**DOI:** 10.1126/sciadv.ads9150

**Published:** 2025-04-09

**Authors:** Raul C. Sîmpetru, Dominik I. Braun, Arndt U. Simon, Michael März, Vlad Cnejevici, Daniela Souza de Oliveira, Nico Weber, Jonas Walter, Jörg Franke, Daniel Höglinger, Cosima Prahm, Matthias Ponfick, Alessandro Del Vecchio

**Affiliations:** ^1^ Neuromuscular Physiology and Neural Interfacing Laboratory, Friedrich-Alexander-Universität Erlangen-Nürnberg, 91052 Erlangen, Germany.; ^2^ Institute for Factory Automation and Production Systems, Friedrich-Alexander-Universität Erlangen-Nürnberg, 91054 Erlangen, Germany.; ^3^ Department of Plastic and Reconstructive Surgery, BG Trauma Clinic, University of Tübingen, 72076 Tübingen, Germany.; ^4^ Querschnittzentrum Rummelsberg, Krankenhaus Rummelsberg GmbH, 90592 Schwarzenbruck, Germany.

## Abstract

Restoring motor function in individuals with spinal cord injuries (SCIs), strokes, or amputations is a crucial challenge. Recent studies show that spared motor neurons can still be voluntarily controlled using surface electromyography (EMG), even without visible movement. To harness these signals, we developed a wireless, high-density EMG bracelet and a software framework, MyoGestic. Our system enables rapid adaptation of machine learning models to users’ needs, allowing real-time decoding of spared motor dimensions. In our study, we successfully decoded motor intent from two participants with traumatic SCI, two with spinal stroke, and three with amputations in real time, achieving multiple controllable motor dimensions within minutes. The decoded neural signals could control a digitally rendered hand, an orthosis, a prosthesis, or a two-dimensional cursor. MyoGestic’s participant-centered approach allows a collaborative and iterative development of myocontrol algorithms, bridging the gap between researcher and participant, to advance intuitive EMG interfaces for neural lesions.

## Teaser

AI-powered EMG decoding with MyoGestic enables intuitive real-time control of spared movements for users with neural lesions.

## INTRODUCTION

Living without a limb’s motor function affects 20.64 million people worldwide with spinal cord injuries (SCIs) (*1*), hundreds of thousands with spinal strokes (*2*, *3*), and 2.23 million with unilateral limb amputations (*4*). Recovering limb function after these injuries remains a crucial challenge. Numerous studies indicate that, despite the lack of visible movements, spared alpha motor neurons can still be voluntarily controlled in patients with motor complete SCI or amputation (*5*–*7*). Recently, we have used surface electromyography (sEMG), a noninvasive neural interface (*8*), to investigate individuals living with SCI who have been clinically labeled as having a motor-complete injury (*9*), and found that these patients can precisely control the activity of several spared motor units (MUs) during intentional attempts to move the hand digits (*5*, *6*).

To date, no EMG-based decoding system can be used to recover limb motor function for multiple types of neural lesions (*10*). Current systems are high-end EMG equipment (e.g., Quattrocento, OT Bioelettronica S.r.l., Torino, Italy; RHD Recording System, Intan Technologies, Los Angeles, USA) that are made for laboratory settings, resulting in recording capabilities of multiple hundred channels (384—Quattrocento; 1024—RHD Recording System), but at the expense of being big and impractical (130 mm by 395 mm by 271 mm—Quattrocento; 170 mm by 420 mm by 600 mm—RHD Recording System) for any daily usage. To counteract this, portable wireless systems have been developed that can record EMG signals in bipolar (*11*, *12*) or monopolar (*13*–*16*) derivation. However, these systems lack the modular software that is needed to interface the spared spinal cord output in an intuitive and effective manner such that myocontrol research may be iterated faster. As a result, current systems are used with custom-made software that can only be operated by experienced researchers and medical professionals (*5*, *6*, *17*, *18*). Current software can only decode a limited number of motor dimensions. The models are either not tailored specifically for individuals with impairments (*11*), or they are designed for specific participants but without any physiological a priori constraints (*19*, *20*) making them less scalable for decoding multiple motor dimensions intuitively for the impaired user.

**Fig. 1.**
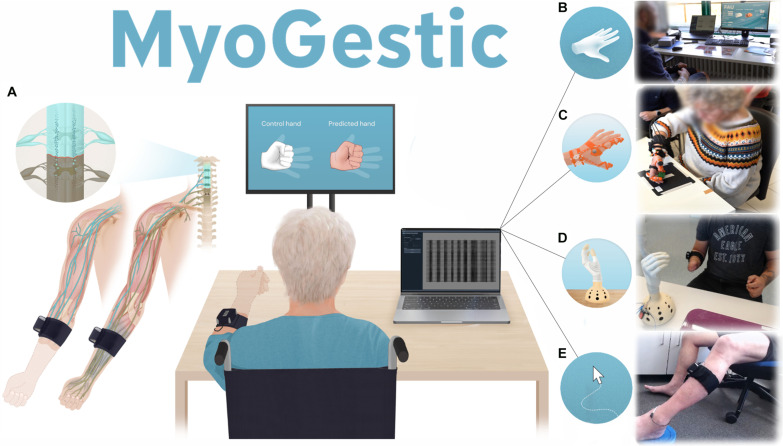
Study overview. (**A**) Four individuals with spinal cord injuries (SCIs) (two with traumatic lesions and two with spinal strokes) and three with amputation (two transradial and one transcarpal) attempted different movements displayed on a screen while their electrophysiological activity was recorded using a 32-channel wireless EMG bracelet. We developed a highly intuitive decoding system that enables participants to control a virtual hand, an orthosis, a prosthesis, or a cursor using their spared neural activity. The framework is highly adaptable to individual needs and was successfully used to decode four motor dimensions in real time, even for a participant who had a motor-complete (AIS B) SCI 21 years ago. The total time from explaining the experiment (to a naïve participant) to controlling four motor dimensions of the hand in real time can take less than 10 min. In a trained participant, it takes less than 4 min to wear the EMG bracelet, train the machine learning model, and control the spared motor dimensions. We further demonstrate that our open-source software is highly adaptable across multiple scenarios. These include the following(from top to bottom): (**B**) three participants with SCI and two with amputations controlling a virtual hand, (**C**) an individual with SCI controlling a custom-made orthosis, (**D**) an individual with an amputation controlling a commercially available prosthetic, and (**E**) a participant with SCI controlling a 2D cursor using their lower limb.

To bridge the gap between research and clinical practice and create a generalizable high-density EMG interface, we developed a lightweight (76 ± 2 g, see Materials and Methods), wireless 32-channel monopolar EMG bracelet with a software framework, MyoGestic, which facilitates rapid adaptability to the needs of impaired participants (Fig. 1A). Our intuitive software (Table 1), capable of both high-performance classification and regression algorithms, allows immediate validation of new hand movements while keeping participants engaged and informed about their progress. The software is integrated with the input data from two digital hands, a hand that is controlled by the user (thereafter the predicted hand) and a control hand that can be used by the experimenter to gather real-time data labels for the machine learning models.

**Table 1.** Time taken to achieve control of one motor dimension using MyoGestic. Each participant was unfamiliar with MyoGestic and was instructed on how to use it using videos and a step-by-step tutorial (see https://nsquaredlab.github.io/MyoGestic, movies S2 and S3) for a maximum of 30 min. Afterward, their time was taken while they were tasked with achieving control of one motor dimension. All participants used MyoGestic on the same machine with a NVIDIA RTX 4090 Laptop GPU.

Participant ID	Background	Sex	Age (years)	Time (min)
User 1	Master’s student (engineering)	F	24	2.20
User 2	Social worker	F	50	2.52
User 3	Master’s student (engineering)	M	24	2.07
User 4	PhD student (engineering)	M	26	2.90
User 5	Postdoctoral researcher (sports science)	M	33	2.23
		Average	30.1 ± 9.9	2.37 ± 0.30 (*32.37 ± 0.30)

*Including 30 min training.

Using the proposed noninvasive neural interface, we were able to decode multiple distinctive gestures in real time from four participants with SCI (*n* = 4 gestures, 2 spinal strokes, and 2 traumatic lesions, Table 2) and three with an amputation (*n* = 5 gestures, 2 transradial, and 1 transcarpal, Table 2 and movie S1). We were able to achieve several controllable motor dimensions (distinct EMG activity patterns that can be used as control signals) for all participants in less than 10 min after explaining the experiment (movies S2 and S3). The decoded motor dimensions could then be interfaced on a display as a controllable virtual hand (Fig. 1B), as input signals for a wearable orthosis/prosthesis (Fig. 1, C and D), or as a two-dimensional (2D) cursor (Fig. 1E).

**Table 2.** Participant characteristics. Age is given in 5-year ranges to protect the participants’ privacy. IL, injury level; ZPP, zone of partial preservation (*9*); AIS, ASIA Impairment Scale (*9*).

Participant ID	Experiment taken part in (all in real time)	Injury type	Time since injury (years)	Age (5-year range)	Sex
SCI 1	Control of a virtual hand (Fig. 3)	Spinal cord ischemia C5/S1/D (IL/ZPP/AIS)	12	55–59	F
	Orthosis control (Fig. 5C)				
	Proportional control (Fig. 5D)				
SCI 2	Control of a virtual hand (Fig. 3)	Spinal cord ischemia C5/S1/D (IL/ZPP/AIS)	2	50–54	M
SCI 3	Control of a virtual hand (Fig. 3 and movie S4)	Traumatic spinal cord injury C6/C7/B (IL/ZPP/AIS)	21	40–44	M
SCI 4	2D foot cursor (Fig. 5B)	Traumatic spinal cord injury L3/S1/D (IL/ZPP/AIS)	3	60–64	M
Amputee 1	Control of a virtual hand (Fig. 4, movie S5)	Transradial amputation	1	45–49	M
Amputee 2	Control of a virtual hand (Fig. 4)	Transradial amputation	0.42	20–24	M
	Prosthesis control (Fig. 5A)				
Amputee 3	Hand digit control (movie S1)	Transcarpal amputation	0.92	35–39	F
	Demonstration of workflow (movie S3)				

This framework could enable researchers to iterate on myocontrol algorithms more quickly, with a participant-centered approach that allows feedback to be integrated immediately and during a posteriori (offline) analysis. By providing already tested code (e.g., real-time plotting or reactive user interface), researchers can dedicate more time to their algorithms and not be concerned with redesigning the experimental setup every time. With a shared visual interface (Fig. 1A), MyoGestic enables a collaborative approach to decoding preserved motor intent. The participant fine-tunes the experimenter’s selections through verbal feedback, creating a dynamic partnership in optimizing functionality. Although longitudinal and clinical tests are needed, our system has the potential to serve as a generalized tool not only for researchers but also for clinicians who may want to train and fine-tune machine learning models for individual patients using commercial assistive devices, thus providing better outcomes for the individual.

## RESULTS

### Design of a universal user-adaptable noninvasive neural interface system

We have developed a noninvasive neural interfacing system that includes a 32-channel monopolar EMG bracelet (Fig. 1A, codeveloped with OT Bioelettronica S.r.l. in Turin, Italy). This bracelet is powered by a commercially available wireless amplifier (MUOVI, OT Bioelettronica) and is complemented by an open-source software framework designed for rapid experimentation and adaptation to participant-specific needs. The system includes two interfaces: one for the participant (Fig. 1A, large monitor) and one for the experimenter (Fig. 1A, laptop screen).

**Fig. 2.**
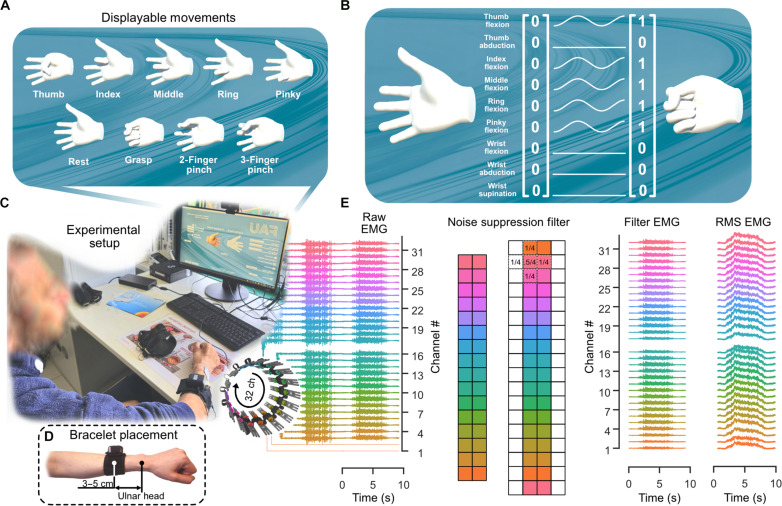
Experimental setup and EMG processing. (**A**) To assist participants in visualizing and maintaining a constant movement speed, we have preset nine distinct movements for display: a resting state, where the hand is in a neutral position with minimal activity, all five individual finger flexions, a hand grasp, and two types of pinches (between thumb and index finger, and between thumb, index, and middle finger). (**B**) The virtual hand state is represented by a 9D vector with values from 0 (rest state) to 1 (fully flexed). (**C**) The experimental setup was kept minimal, comprising a laptop running MyoGestic, an EMG bracelet, and a monitor. This design allowed the experiment to be conducted conveniently in different locations, such as hospitals (as exemplified here) or in our laboratory environment. The monitor showed two hands. The white “control” hand controls the participant’s pace by demonstrating any of the preset movements discussed in (A). Simultaneously, the predictions generated by our model are shown on the “predicted” hand at 32 Hz. This frequency ensures smooth transitions between movements, mitigating the uncanny valley effect. (**D**) The 32-channel dry monopolar EMG bracelet was positioned for patient convenience at 3 to 5 cm from the distal ulnar head. The ground reference was placed on the nearest accessible bony structure, such as the ulnar head or elbow. (**E**) EMG processing pipeline example using data from a bracelet with 2 electrodes that had no skin contact (1 and 17). The initial EMG matrix is reshaped to represent the bracelet as the physical 16-row, two-column structure. Row-wise circular padding simulates the looping of the bracelet, followed by column-wise zero padding for applying a 3 × 3 high-frequency noise suppression kernel using convolution. Last, one root mean square (RMS) value is computed per channel.

Individuals who suffered from chronic hand function loss (*5*, *6*, *17*) often struggle to imagine and attempt hand movements, which imposes a challenge in recalling or learning new neural commands necessary for execution. To address this challenge, we designed the participant interface featuring two hands (Fig. 1A, large monitor): one white hand guiding participants (the control hand) and one light skin-toned displaying their predicted movement (the predicted hand). This setup aided the participants in conceptualizing and maintaining a consistent frequency of specific hand movements that can be defined by the experimenter. By decoding the EMG activity generated by the user attempting the observed motion of the control hand, we allowed the participants, using the prediction hand, to intuitively attempt the natural motor commands that once controlled the movement of the hand (*6*) (Fig. 1B). We predefined nine hand movements for our experiments (Fig. 2A): resting state with all digits extended, individual finger flexions, grasp, two-finger pinch (thumb and index), and three-finger pinch (thumb, index, and middle). The state of each hand at any given time is represented by a 9D vector, whose elements control a simplified joint model of the hand (Fig. 2B, see Materials and Methods). This design choice enables straightforward definition of novel hand movements as well as rapid and accurate tracking of ongoing motions.

The lightweight (76 ± 2 g) EMG bracelet was fabricated from a flexible printed circuit board, featuring dry gold-coated copper electrodes housed within a fabric sleeve (Figs. 1A and 2C; see Materials and Methods). Five bracelet lengths (18, 19, 21, 23, and 33 cm) were available, with the one closest to the participant’s limb circumference selected to ensure optimal EMG signal quality and minimal electrical noise interference. The selected bracelet was placed about 3 to 5 cm away from the wrist (distal ulnar head, Fig. 2D). An adhesive ground reference electrode was attached to the elbow. The signals were recorded in real time at 2000 Hz and streamed as 111 nonoverlapping EMG windows per second using a commercially available wireless amplifier (see Materials and Methods).

To achieve the highest possible prediction rate (111 Hz) and demonstrate the potential of wearable neural interfaces for individuals with hand motor impairments, we limited our signal processing to only what was necessary. The EMG signals were filtered to remove high-frequency noise such as movement artifacts, followed by computation of the root mean square (RMS) value for each channel within each window (Fig. 2E; see Materials and Methods). We selected the CatBoost algorithm (*21*), an established model within the machine learning community, for the classifier, as it has been shown to excel among its peers in terms of performance, and benefits from being open sourced under an Apache 2.0 license. Moreover, the user can also choose to predict simultaneously and proportionally with a custom-made convolutional neural network from the raw monopolar EMG signals (*22*, *23*).

Our interface, used to orchestrate the recording, training, and validation of the decoded motor dimensions, strikes a balance between streamlining processes and providing programmable freedom. Each step—connecting to the device, recording, and training/validation—is separated into panels that can be viewed one at a time, minimizing cognitive load during the experiment and reducing potential errors (movies S2 and S3). The interface was designed so that the user navigates from top to bottom within each panel and from left to right to switch between panels. Regardless of the current step, we display all the EMG channels in real time (111 Hz), allowing us to monitor signal quality and intervene if necessary (e.g., addressing issues with bracelet slipping of the arm stump). The design minimizes the time it takes for participants to receive feedback, keeping them informed throughout each stage. Movie S3 shows the real-time workflow from recording to controlling three motor dimensions on a transcarpal amputee. We then assessed the usability and intuitiveness of the MyoGestic interface. For this purpose, we asked five naïve users (two women and three men with an average age of 30.1 ± 9.9 years; Table 1) to learn MyoGestic using the same machine with an NVIDIA RTX 4090 Laptop GPU. The participants were instructed through the video tutorials and a step-by-step documentation (see https://nsquaredlab.github.io/MyoGestic, movies S2 and S3) for a maximum duration of 30 min. In these 30 min, the participants were also instructed on how to don the EMG bracelet and connect it to the computer. After 30 min, all participants were asked to control one motor dimension on an uninjured participant (e.g., opening and closing the virtual hand). The average time taken to achieve this (after the 30-min training session), was 2.37 ± 0.30 min (Table 1).

### Motor intent decoding from individuals with SCI

Using our neural interface system, we recorded data from three participants with cervical (C5 to C6) SCI (Table 2; see Materials and Methods). Participants 1 and 2 experienced a spinal ischemia (stroke), while participant 3 had a traumatic lesion. The participants were seated in front of a monitor and received a 10-min introduction of the virtual hand interface (Fig. 2, A to C) while the proposed EMG bracelet was fitted (Figs. 1A and 2, C and D, and fig. S1A) and the signals were visually inspected for noise caused by issues such as poor electrode-skin contact.

**Fig. 3.**
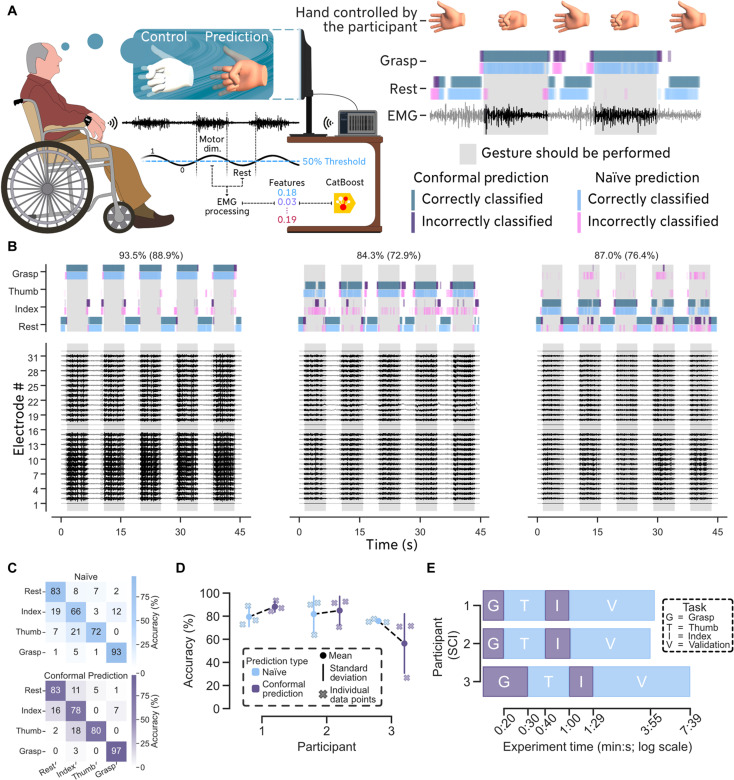
Decoding of hand motor dimension for individuals with SCI. (**A**) Three participants with SCI (Table 2) were asked to follow three different movements shown on a screen. Their forearm EMG activity was streamed using a 32-channel bracelet and processed by MyoGestic. We validated our system by asking the participants to follow the same trained movements again in real time. To counteract EMG signal variability, we embedded in MyoGestic a conformal prediction algorithm to quantify and resolve prediction uncertainties. The participants followed a sinusoidal movement for each motor dimension for 20 s (30 s for the third participant). To increase rest-state detection robustness despite the prevalence of involuntarily active MUs, a movement was defined as performed when the guiding hand surpassed the 50% flexed state; otherwise, it was considered as rest state. Movie S4 shows the third participant performing the experiment. (**B**) Exemplary real-time prediction and EMG signals of the first participant with SCI. The attempts at controlling the three movements are displayed across individual panels for clarity and convenience. The accuracy achieved with conformal prediction is displayed at the top of each movement, with the naïve approach shown in parentheses. (**C**) Confusion matrix for the data presented in (B). (**D**) Classification accuracy for each participant using the naïve approach and conformal prediction (see Materials and Methods). The accuracies for the participants were 79.4 ± 8.4%, 81.8 ± 15.6%, and 75.8 ± 1.5% for the naïve approach, and 88.3 ± 4.7%, 84.9 ± 12.4%, and 56.4 ± 25.7% using conformal prediction. (**E**) The time taken for each participant to record and validate four motor dimensions was 4 min 12 s, 3 min 55 s, and 7 min 39 s. We asked each participant to execute each movement for 20 s, and to perform six repetitions within a 45-s span for validation.

The next 15 min were dedicated to identifying the hand movements that the participants could reliably recall after years since the lesion (12, 2, and 21 years, respectively) and living with hand paralysis (Fig. 3A). During each movement attempt, we visually screened the EMG signals for voluntary changes. After identifying three different movements in addition to the rest state that were controllable, we visualized, recorded, and trained the machine learning model with their data using MyoGestic (Fig. 2, A to C, and fig. S1A) for 20 s per motor dimension. Participant 3 requested that their data recording duration to be adjusted to 30 s per movement, for personal preference. The recordings involved participants attempting to execute the movement displayed by the control hand. In this setup, the EMG signals served as input while the virtual hand positions acted as ground truths. We previously observed that individuals with SCI (*6*, *18*), when attempting to move, could activate non–task-modulated MUs that affect the rest state. The MUs are likely caused by maladaptation of the motor neuron intrinsic inputs (*24*). To address this issue, the sinusoidal activity of the virtual hand was divided at a 50% amplitude threshold (Fig. 3A). EMG activity exceeding this threshold was classified as the active state, while activity below the threshold was classified as resting state.

Using these data, we trained a CatBoost (*21*) classifier model for each participant to decode the four motor dimensions. All three participants were able to produce voluntary neural signals for grasping, flexing the thumb, and flexing the index finger individually.

To validate the trained classifier, we asked each participant to control the previously executed movements in real time. Participants followed the guiding hand (Figs. 1 and 2A), with model predictions displayed as a light skin-toned hand (Figs. 1 and 3A). Each participant was asked to follow each motor dimension for six repetitions within 45 s, although participant 3 opted to do more repetitions for personal preference.

Figure 3B shows the predictions using both an uncertainty-unaware (naïve) approach and an uncertainty-aware approach (conformal prediction; see Materials and Methods), along with the synchronous EMG signals for the first participant (see Fig. 3C for confusion matrix). Briefly, we used conformal prediction to gauge prediction uncertainty, which we anticipated might happen due to the participants having involuntary MU activity. Using the uncertainty estimation, the predictions of the machine learning model could be made more stable by disregarding uncertain ones. For example, this safety feature helps the prevention of involuntary openings of the hand while attempting to grasp, but it may also reduce the user’s reaction speed because the model must be certain that opening the hand is the intended action (Fig. 3C). Results for the other two participants are provided in Fig. 3D and fig. S2A. Movie S4 shows the third participant during their attempt at controlling four motor dimensions in real time. The participants achieved accuracies of 79.4 ± 8.4%, 81.8 ± 15.6%, and 75.8 ± 1.5% using the naïve approach, and 88.3 ± 4.7%, 84.9 ± 12.4%, and 56.4 ± 25.7% using conformal prediction (Fig. 3D). Notably, the third participant (21 years living with a motor-complete SCI, Table 2) had lower accuracy with the conformal prediction than with the naïve approach.

The time taken to record movements and validate control over the trained motor dimensions was 4 min 12 s, 3 min 55 s, and 7 min 39 s for each participant, respectively (Fig. 3E).

### Motor intent decoding from individuals with a transradial amputation

We also recorded data from two individuals with transradial amputation (Table 2; see Materials and Methods) using our neural interface system. Similar to the participants with SCI, our first goal was to determine how many motor dimensions could be decoded from the bracelet signals in real time and to assess their decoding speed. For the participants with SCI, we focused on four motor dimensions (resting state plus three movements). In contrast, we asked the individuals with transradial amputation to attempt to control each digit individually.

**Fig. 4.**
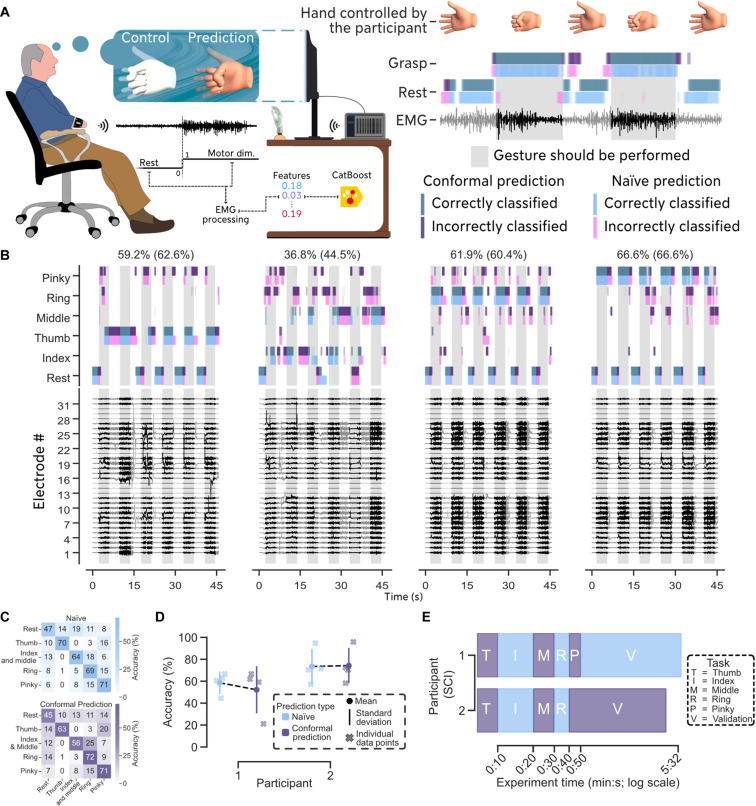
Decoding of hand motor dimensions for individuals with a transradial amputation. (**A**) Two participants with a transradial amputation (Table 2) were asked to flex all their fingers while being visually guided by a hand displayed on a screen. Using the proposed 32-channel bracelet, their forearm EMG activity was streamed and processed by our software, MyoGestic. We validated our system by having the participants repeat the same trained movements in real time. To address EMG signal variability, we applied conformal prediction (see Materials and Methods) to quantify and resolve prediction uncertainties. The participants were instructed to hold the fully flexed state of each finger for 10 s. Movie S5 shows the first participant performing the experiment described. (**B**) Exemplary real-time prediction and EMG signals of the first individual with an amputation. The attempts at controlling the four movements are displayed across individual panels for clarity and convenience. The accuracy achieved with conformal prediction is displayed at the top of each movement, with the naïve approach shown in parentheses. (**C**) Confusion matrix for the data presented in (B). (**D**) Classification accuracy for both amputees using the naïve approach and conformal prediction (see Materials and Methods). The accuracies for the participants were 58.5 ± 9.7% and 73.5 ± 15.6% for the naïve approach, and 52.2 ± 21.0% and 74.2 ± 15.7% using conformal prediction. (**E**) Time taken for each participant to record and validate five motor dimensions was 6 min 4 s and 5 min 42 s. We asked each participant to execute each movement for 10 s and to perform six repetitions within a 45-s span for validation.

For the participants with amputations, we recorded only the end state of each motor dimension for 10 s (Fig. 4A) because reaching a steady rest state posed no challenge for them compared to the participants with SCI. Both participant populations could differentiate between rest and four other movements. The first participant with a transradial amputation could not reliably produce different EMG signals to differentiate between the index and middle finger. The second amputee could not voluntarily control the pinky finger and could not imagine the thumb flexing independently of the index finger. For the thumb, we remapped intended thumb flexion as extension. In summary the first amputee could control the thumb, the index and middle fingers together, the ring finger, and the pinky. The second participant with a transradial amputation could control every digit except the pinky.

Figure 4B shows the predictions synchronized with the EMG signals of the first amputee (see Fig. 4C for confusion matrix). Data from the second participant can be found in Fig. 4D and fig. S2B. Movie S5 shows the first participant with a transradial amputation controlling five motor dimensions in real time. Figure 4D shows the accuracy of the two individuals with an amputation both with (52.2 ± 21.0% and 74.2 ± 15.7%) and without uncertainty awareness and solving (58.5 ± 9.7% and 73.5 ± 15.6%). The time taken to achieve five motor dimensions was 6 min 4 s and 5 min 42 s, respectively (Fig. 4E).

### Adaptability to different experimental scenarios: Bionic limb, active exoskeleton, multidimensional cursor control, and gaming

**Fig. 5.**
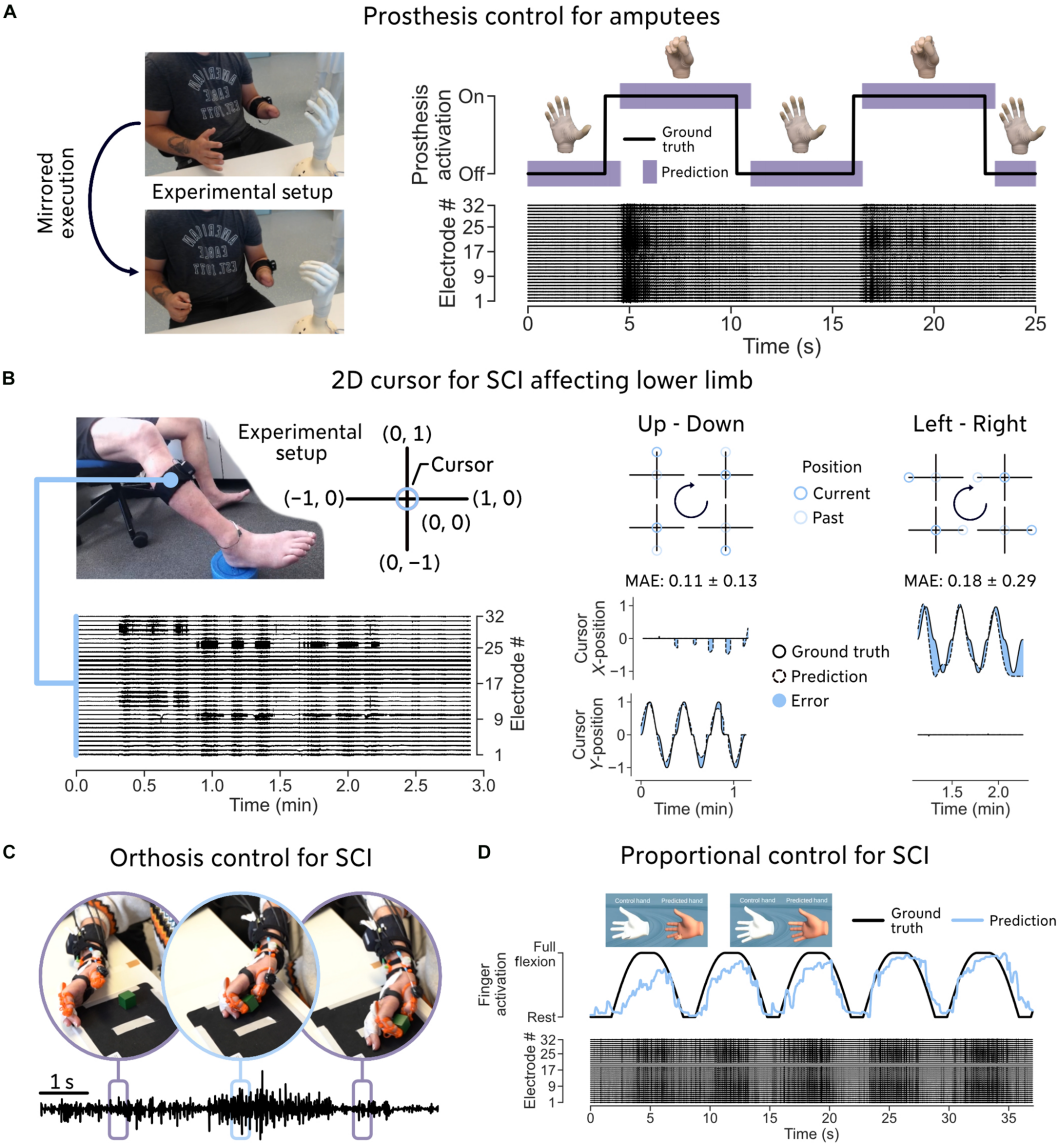
Demonstration of MyoGestic’s adaptability to different experimental scenarios. Our software framework, MyoGestic, is designed to be agnostic to input, algorithm, and output. For user convenience, we maintain the EMG bracelet while varying the output system (A to C) and algorithms (D) to demonstrate its capabilities. (**A**) The second participant with a transradial amputation was asked to control the three-finger pinch of the Michelangelo Hand prosthesis (Ottobock, Duderstadt, Germany). The participant was instructed to perform the hand-closing motion with both hands for visual confirmation of the movement. To guide the participant, a visual interface was used (Fig. 2B). (**B**) A fourth individual with SCI (Table 2) was recruited to control a 2D cursor using the neural activity from their spared leg. The EMG bracelet was placed around the bellies of the calf muscles. The participant was instructed to perform inversion-eversion (left-right) and dorsiflexion-plantarflexion (up-down) movements, always pausing briefly in a natural rest position. (**C**) The first participant with SCI was also outfitted with a custom-built orthosis [see Walter *et al.* (*25*) for design details] and asked to perform the box and block test. (**D**) Last, we implemented a deep neural network (see Materials and Methods) to proportionally predict the control of a motor dimension that the first participant with SCI could best remember and envision.

**Fig. 6.**
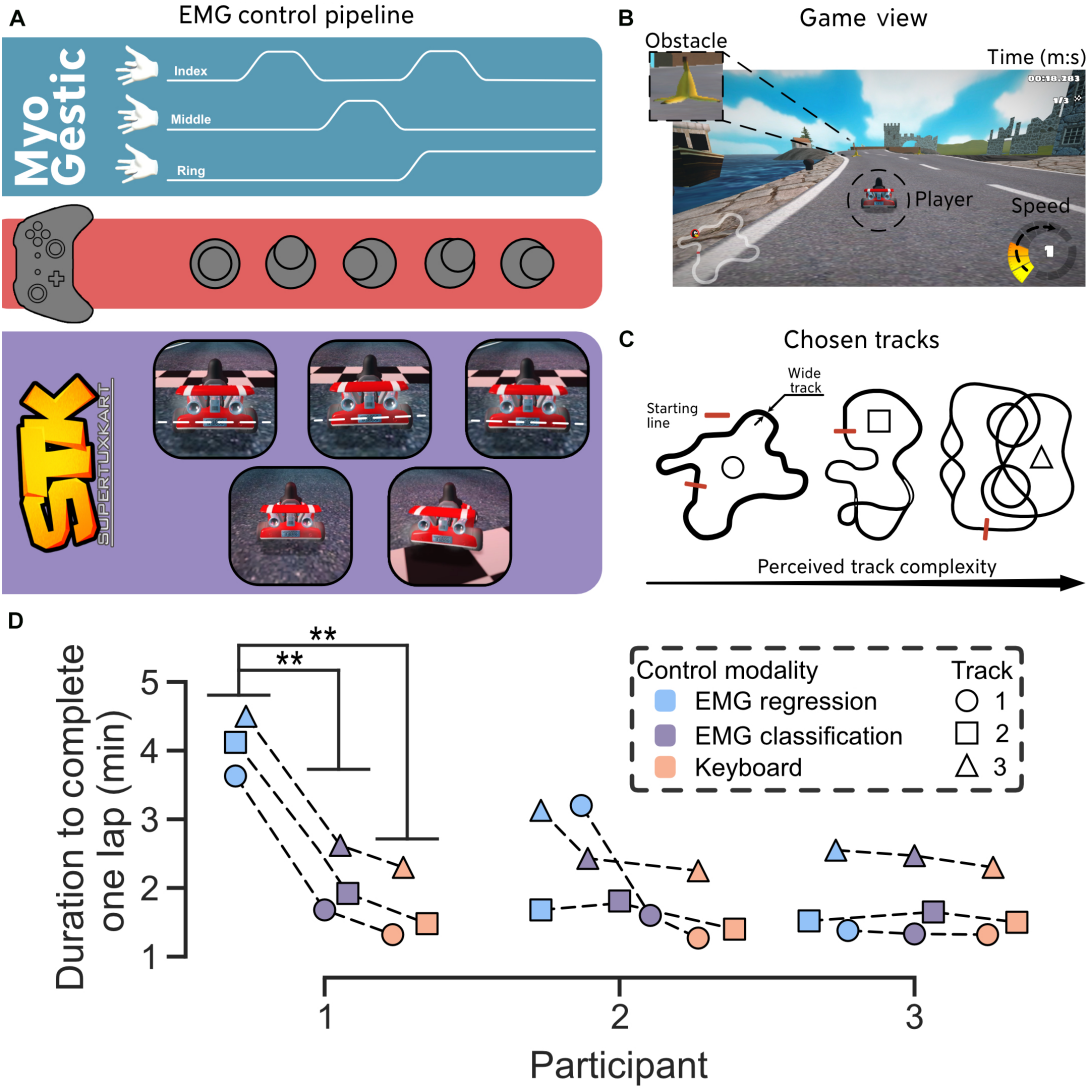
Demonstration of MyoGestic’s adaptability for virtual interactions. Three able-bodied participants (males, 29.7 ± 6.2 years old) were asked to play an open-source racing game, SuperTuxKart (*26*), using a regression and a classification model implemented in MyoGestic. The participants had no prior knowledge of the game or the control schemes, except for the recording of the training data that occurred approximately 15 min before they played the game. (**A**) The regression model was trained for each participant to output three continuous values, each ranging from 0 to 1, representing the flexion of the index, middle, and ring fingers. These values were converted into forces applied to a simulated analog controller. The index finger controlled the acceleration, while the middle and ring fingers controlled the left and right directions, respectively. For the classification approach, acceleration is always set to maximum, with the index finger steering left and the ring finger steering right. (**B**) Participants were asked to complete one lap per track as quickly as possible, avoiding obstacles like banana peels. Power-ups such as nitro or boosts were not used. (**C**) Three tracks of increasing complexity—“Nessie’s Pond” (wide roads, no shortcuts), “Ravenbridge Mansion,” and “Volcan Island” (narrow roads, shortcuts)—were selected. (**D**) Completion times for one lap using the EMG-based controllers (no prior experience at track 1 start) and a keyboard were recorded. Time differences between the EMG regression scheme and the keyboard were 2.38 ± 0.18, 1.03 ± 0.68, and 0.10 ± 0.08 min for participants 1, 2, and 3, respectively. For the EMG classification scheme, differences were 0.37 ± 0.05, 0.31 ± 0.10, and 0.10 ± 0.06 min. A significant difference (**P < 0.01; Welch’s *t* test) was found for participant 1 between the regression and classification schemes and between the regression and keyboard control.

To demonstrate the adaptability of our software framework, we tested it in six different and realistic scenarios that could be of interest to the myocontrol research community (Fig. 5—scenarios 1 to 4, movie S1—scenario 5, and Fig. 6—scenario 6).

We integrated the Michelangelo Hand prosthesis (Ottobock, Duderstadt, Germany) as an output system into MyoGestic. One of our participants with transradial amputation controlled the bionic hand through MyoGestic in an effective and intuitive way (Fig. 5A). The prosthesis was placed on a socket in front of the participant (Figs. 4A and 5A). The participant was asked to follow the virtual guiding hand (Figs. 1 and 2A), which showed a continuous opening and closing of a three-finger pinch, and to execute the displayed movements bilaterally for visual confirmation of execution.

Moreover, a participant with an SCI affecting the lower limb (Table 2) was recruited to control a 2D cursor with five motor dimensions (Fig. 5B). For this, we developed a larger EMG bracelet (33 cm) to record the activity of the calf muscles. We recorded the rest position, as well as the maximum possible inversion, eversion, dorsiflexion, and plantarflexion of the foot. Each movement was recorded starting from and returning to the rest position. For real-time cursor testing, we divided the movements into left-right and up-down directions to reduce cognitive strain on the participant. The movements always included a brief stop in the rest position before continuing in the opposite direction.

For the Michelangelo Hand, we relied on a preexisting communication protocol. However, we also implemented a connection from scratch for a custom-built hand orthosis [see Walter *et al.* (*25*) for details]. We asked the first spinal cord injured participant to use the orthosis to attempt the box and block challenge (Fig. 5C). In addition, we integrated a custom-built deep neural network (see Materials and Methods) into MyoGestic to predict hand position proportionally rather than just classifying the start and end states. Figure 5D shows the first participant with SCI attempting a proportional pinky movement. We chose the pinky finger because it was easier for the participant to proportionally control the EMG activity by attempting pinky movements. We further recruited an individual with a transcarpal (forearm muscles are preserved) amputation (Table 2) to examine the possibility of decoding the motor intent of each finger (movie S1).

Last, we asked three able-bodied participants (males, 29.7 ± 6.2 years old) to play an open-source racing game, SuperTuxKart (*26*), using two deep learning models based on the same architecture applied in Fig. 5D. The first model was trained to predict the independent flexion of the index, middle, and ring fingers proportionally (Fig. 6A). Each flexion output, constrained between 0 and 1, was applied as a force on a simulated analog joystick controller for input into SuperTuxKart (Fig. 6A). Participants were instructed to complete one lap on each track as quickly as possible, avoiding obstacles and without using any power-ups (Fig. 6B). Three different tracks (“Nessie’s Pond,” “Ravenbridge Mansion,” and “Volcan Island”) were selected to increase the perceived complexity. Track 1 featured wider roads and no shortcuts, whereas tracks 2 and 3 included shortcuts (Fig. 6C). The second model used a simplified classification-based control scheme, where the index finger steered to the left, the ring finger steered to the right, and acceleration was continuously active. We recorded the time each participant required to complete one lap using both EMG-based controller schemes (no prior experience at the start of track 1), and a second attempt with a keyboard. These times are displayed in Fig. 6D. All participants successfully completed each track. The differences in completion times between the EMG-based regression control scheme and the keyboard were 2.38 ± 0.18, 1.03 ± 0.68, and 0.10 ± 0.08 min for participants 1, 2, and 3, respectively. For the EMG classification control scheme compared to the keyboard, the differences were 0.37 ± 0.05, 0.31 ± 0.10, and 0.10 ± 0.06 min respectively. A statistically significant difference (**P < 0.01; Welch’s *t* test) was observed for participant 1 between the regression and classification schemes, as well as between the regression scheme and keyboard control.

These tests suggest that MyoGestic provides an intuitive and flexible machine learning framework to efficiently control hand motor functions. Through tailored artificial intelligence (AI) models, individuals can quickly and intuitively map these functions to any required output modality.

## DISCUSSION

Our study demonstrates the potential of our neural interface system called MyoGestic to decode motor intentions in two individuals with traumatic SCI, two with stroke, and three with amputations. The shared visual interface between the participant and experimenter creates a collaborative environment where the experimenter’s AI training choices can be complemented by the participant’s feedback, maximizing motor recovery.

### Motor intent decoding from individuals with SCI

We show that one participant with cervical SCI and two with spinal stroke (Table 2) could execute in real time distinct EMG patterns for grasping, flexing the thumb, and the index finger (Fig. 3), despite long-term paralysis. This finding reconfirms previous studies (*5*, *6*, *17*, *18*) indicating the presence of spared motor unit encoding hand movements even after SCI and prompts a reevaluation of current SCI assessment standards (*9*), suggesting they may need to be extended to incorporate EMG signal evaluation.

In our previous work, we demonstrated that a deep-learning system could identify which movements are proportionally decodable in participants with motor-complete SCI (*18*). We proposed that such techniques could enhance injury assessment by distinguishing between EMG-complete and EMG-incomplete statuses. This study builds on our previous research by showing that voluntary signal assessment can be accomplished in under 10 min (Fig. 3E) using comparatively simple algorithms (see Materials and Methods). Our open-source framework, MyoGestic (Fig. 2 and fig. S1A), could thus aid medical professionals in determining the presence of voluntary EMG signals in individuals with SCI. This capability not only has the potential to positively influence the psychological state of individuals affected but also supports researchers and medical professionals in designing and selecting the most suitable assistive devices.

### Motor intent decoding from individuals with a transradial amputation

We tested our neural interfacing system on two individuals with a transradial amputation and found that the EMG signals had some artifacts (Fig. 4B), primarily because our bracelets, although somewhat form compliant, were not perfectly tight around the participants’ stumps. This issue could be resolved by tailoring a participant-specific bracelet with distinct sizes and shapes.

Despite this challenge, we demonstrated that we could decode and validate five motor dimensions in under 10 min (Fig. 4) using MyoGestic. In addition, the typical control paradigm for prosthetics involves using muscle cocontractions (coactivations of both agonist and antagonist muscles) to change the selected movement patterns, which is not intuitive for the user. MyoGestic allows for intuitive control by enabling the user to directly attempt a specific movement with the spared neural structures, e.g., “actually grasp and not just initiate the grasping action by cocontracting” (reported by one of our participants).

### Generalizable control

MyoGestic was designed as a versatile interface to connect the motor intent of participants with various output modalities. Our study demonstrated that six participants, three with SCI and three with amputations (Table 2), successfully controlled a virtual hand (Figs. 3 and 4).

In addition, we showed six different realistic use cases of MyoGestic:

1) Connecting to a commercially available system using a preexisting communication protocol (Fig. 5A),

2) Interfacing the lower limb as opposed to the upper one and outputting the decoded motor dimensions as a 2D cursor as opposed to a virtual hand (Fig. 5B),

3) Interfacing with a custom-built system and thus requiring a new implementation of a communication protocol from scratch (Fig. 5C),

4) Integrating a new algorithm that can do regression instead of classification (Fig. 5D),

5) Controlling all hand digits using the preserved forearm muscles after amputation (movie S1), and

6) Controlling an open-source racing game using an EMG-based regression and classification scheme (Fig. 6).

Achieving intuitive myocontrol heavily depends on the decoding algorithm, as it directly influences user satisfaction and device reliability. MyoGestic was designed to allow researchers to select and modify algorithms as needed to accommodate individual injuries and unique challenges. A key challenge is determining the number of motor dimensions preserved after the injury. While we have previously used deep learning models to detect preserved functions in participants with SCI (*18*) and could leverage medical records to identify preserved muscles in participants with amputation, it remains uncertain if individuals can recall the activation patterns of their retained anatomy. In addition, physiological factors, such as motor unit rotation (*27*) and fatigue, add complexity by potentially creating different input signals for the same motor intent. It should also be noted that the users had no prior experience with any of the control schemes used in our experiments, except for the brief training session used to train the models a few minutes before attempting the experiments in real time.

From a usability perspective, we found that classification (Figs. 3, 4, and 5, A to C) is often preferable for individuals with neural lesions, as the detection algorithm can be fine-tuned to operate effectively with low force levels. This tuning allows users to perform various movements without experiencing fatigue—an essential factor, particularly for those who have experienced years of hand function loss. Proportional control (Figs. 5D and 6), which correlates with exerted force, also offers valuable functionality. However, because our experiments involved minimal participant training, we observed that participants were more prone to fatigue with proportional control as it inherently requires greater cognitive and physical effort, introducing more variability in the output data (*22*). To further reduce signal variability, we used conformal prediction (Figs. 3C and 4C) to detect and reject uncertain predictions in the classification tasks. All participants reported preferring this control scheme, as the system maintained the current state longer until a clear new motor intent was detected. This occasionally resulted in movements starting or ending slightly later than expected, but it contributed to overall control stability. Two out of three participants with SCI (Fig. 3D) and one out of two with amputations (Fig. 4D) improved their control when uncertain predictions were removed.

MyoGestic was therefore designed as a universal interface between users and various output devices, allowing myocontrol researchers the freedom to implement diverse control schemes. This aims to provide individuals with neural lesions with the capability to control different assistive devices, ultimately improving their quality of life. Future research should explore the extension of MyoGestic’s capabilities to help other relevant problems, such as integrating with neuroorthoses for enhancing or restoring mobility.

### Comparison with commercial hardware standards and available EMG software

It is important to highlight the difference between our system and commercial assistive devices. Commercial assistive devices are tailored to the shape and size of the patient anatomy, while our hardware was not. To accommodate a broad participant population, we produced five bracelet sizes (18, 19, 21, 23, and 33 cm). This is particularly important for amputees, as our bracelets cannot perfectly conform to the preserved anatomy, resulting in noisy channels due to suboptimal skin-electrode contact (Fig. 4B). For optimal results, each participant should ideally be measured, and a custom bracelet should be designed for them. A custom fit would likely improve the accuracy and reliability of the EMG signal recordings. However, most assistive devices use a low number of electrodes (<10), which, as our previous research (*18*) shows, negatively affects hand kinematic predictions. In contrast, our system uses 32 electrodes, allowing it to decode richer patterns and achieve an unprecedented level of motor dimensions (Figs. 3 and 4). The last noteworthy difference is the lack of dedicated learning time with our neural interface for the participants. Unlike most assistive devices that necessitate extensive physiotherapy and rehabilitation sessions to master the system, our participants did not have such preparatory sessions, in which potentially more data can be recorded per movement compared to our maximum of 30 s. While the lack of training could affect the participants’ proficiency in generating clear and consistent EMG signals, potentially affecting the system’s performance, it also demonstrates that executing intuitive movements is preserved even after years of limb function loss. We hypothesize that the limited data recorded in this study may not be sufficient for achieving reliable longitudinal control. This insufficiency could arise from physiological changes, such as fatigue, motor unit rotation (*27*), and maladaptation at the motor neuron level (*24*), as well as external factors like temperature and humidity. Future research should conduct longitudinal studies to determine the extent of these influences on reliability and explore methods for anticipating or mitigating such changes.

From a software perspective, there are several commercial (e.g., OT BioLab+ by OT Bioelettronica S.r.l., Turin, Italy; myoRESEARCH by Noraxon USA, Scottsdale, AZ) and open-source [e.g., LibEMG (*28*) and AxoPy (*29*)] EMG-based solutions available. Most of these focus primarily on offline recording and processing of EMG signals, with limited real-time control capabilities (e.g., plane game in OT BioLab+ or snake game with LibEMG). In contrast, MyoGestic is designed specifically for real-time interaction and immediate feedback—essential features for adaptive control in wearable technologies. Moreover, MyoGestic uniquely offers a ready-made physiological control model, addressing the needs of individuals with neural lesions through its virtual hand interface (Fig. 1A). This interface builds on the concept of human-to-human communication, providing a shared visual space for both the experimenter and participant. The AI model adapts based on the data selection of the experimenter and can be fine-tuned based on the feedback given by the participant, creating a collaborative setup aimed at restoring motor function. Combined with our high-density EMG bracelet, this approach enables control of multiple motor dimensions shortly after donning the system. MyoGestic’s open-source code base allows researchers to integrate any EMG recording device of their choice and combine it with other software solutions.

## MATERIALS AND METHODS

### Study design

We recruited two participants with an SCI, two with stroke, and three with an amputation (Table 2). Participants gave their informed written consent to participate in the study. The study was carried out in accordance with the Declaration of Helsinki. All procedures and experiments were approved by the ethics committees of the Friedrich-Alexander-Universität Erlangen-Nürnberg (applications 22-138-Bm and 21-150-B) and of the Eberhard-Karls-Universität Tübingen (applications 470/2019B02 and 181/2020B01). Before the experiments, the participants received a detailed explanation of the study again and were asked to reaffirm their consent to participate.

Each experiment was conducted in a single session per participant. The initial 10 min were allocated for hardware setup and introduction to the virtual hand interface (Figs. 1 and 2) or the 2D cursor for the leg experiments (Fig. 5B). For participants with an SCI, the bracelet was placed 3 to 5 cm above the distal ulnar head for the participants’ convenience (Fig. 2C). This position was chosen because it replicated the position of a wristwatch, something most participants use daily, thus minimizing the need for adjustment. We placed the bracelet on the individuals with an amputation as close as possible to the site of amputation on the remaining muscle bellies under the guidance of the presiding medical professional. For the fourth participant with SCI, we placed the bracelet on the bellies of the calf muscles (Fig. 5B). Five bracelet lengths (18, 19, 21, 23, and 33 cm) were available, with the one closest to the participant’s forearm (or leg) circumference selected to ensure optimal EMG signal quality and minimal electrical noise interference. An adhesive ground reference electrode (Adhesive electrode 4 cm by 4 cm, axion GmbH, Leonberg, Germany) was attached to the elbow or the medial malleolus for the leg.

The next 15 min were dedicated to identifying the hand (foot for SCI 4) movements the participants could reliably recall after years of living with motor function loss. The following movements could be displayed on a screen in front of them as a virtual hand to provide visual aid (Fig. 1, middle): (i) thumb flexion, (ii) index flexion, (iii) middle flexion, (iv) ring flexion, (v) pinky flexion, (vi) grasp, (vii) thumb and index pinch, and (viii) thumb, index, and middle finger pinch.

For the foot, we used a red circle that could move up, down, left, and right as a 2D cursor (Fig. 5B). We used MyoGestic’s real-time plotting capabilities to visually inspect the participants’ recalled movements, ensuring that they were distinguishable from each other. Our primary goal was to maintain intuitiveness by having participants attempt to execute and be able to display the same executed movement on the screen. However, we remained adaptable in case the prepared movements were not recallable or distinguishable. MyoGestic allowed us to map the executed movements to alternate ones for display without any code changes. For example, if a participant recalled how to extend their fingers instead of how to flex them, we could use this as a proxy for grasping. In this case, the grasping action would still be accurately displayed in the flexing state (Fig. 1, middle).

After identifying different movements (three for SCIs and four for amputations) that are both visually distinguishable and reliably recallable by the participant, we displayed each of the said movements in a sinusoidal pattern (relaxed state to fully executed) on the display at a speed of 1 execution per 5 s with 1.5 s hold time for both the rest phase and the executed phase. The EMG together with the movement kinematics were recorded for 30 s per movement. To counteract the prevalence of involuntarily active MUs, which, when unattended, results in poor rest-state behavior, we separated every movement task into an active state and a rest state. This ensured that the rest state could be reached even if non–task-modulated MU activity remained long after the movement was executed, and the participant’s hand returned to a resting position. We chose 50% movement activation for the class boundary. In total, the rest state had 45 s of data, while every other movement had 15 s each.

### Bracelet

The EMG bracelet consisted of 32 dry gold-coated copper electrodes spread over 2 columns with 16 rows. Column-wise, the interelectrode distance (IED) is fixed at 2 cm, while row-wise, it varied between 1 and 1.5 cm depending on the bracelet length selected and the circumference of the forearm due to the stretchable printed circuit board. The bracelet together with the amplifier weighed 76 ± 2 g (measured using a kitchen scale, OK. OKS 3220). Exact measurements can be found in fig. S3.

The raw signals from the electrodes were sampled at 2000 Hz, amplified with a gain of 4, digitized to 16 bits, and streamed at approximately 111 Hz by a commercially available wireless amplifier (MUOVI, OT Bioelettronica S.r.l., Turin, Italy). The amplifier thus could stream 111 nonoverlapping EMG matrices (32 channels × 18 samples) per second of data via a Transmission Control Protocol connection.

### Visual interface

The visual interface built using Unity 2021.3.20f1 (Unity Technologies, San Francisco, USA) displayed two hands: a control hand, which controlled the user’s movement pace, and a predicted hand, which provided real-time feedback on the movements attempted by the participants (Fig. 1). The hand model (HTC VIVE Unity SDK, HTC Corporation, New Taipei, Taiwan) was animated using a bone rig that allows each hand joint to be represented by a quaternion.

To aid the participants in imagining and following a movement, the control hand could display nine distinct hand movements: a rest state, individual digit flexions, a grasp, and a two-finger (thumb and index) or three-finger (thumb, index, and middle) pinch. The frequency and holding time for both extrema (resting and full flexion) could be adjusted to best accommodate each participant. For our experiments, we set the holding time to be 1.5 s, resulting in one movement cycle (resting to full flexion and back) per approximately 7.5 s. The control hand was represented in any state by a 9D vector with values ranging between 0 and 1 (Fig. 2B). The rest state was represented by a vector of nine zeros. The first two values control thumb flexion and abduction, respectively. The next four values control the flexion of the index, middle, ring, and pinky fingers. The last three values represent wrist flexion, adduction, and pronation. During recording, both for training and for validating the model, the control hand state was streamed and saved at 60 Hz using a User Datagram Protocol (UDP) connection.

The prediction hand could also be represented by the same 9D vector structure to provide feedback to the participant. For the classification approach in this work, we use linear interpolation between the quaternions described by the previous and new vector states to create a smooth transition rather than abruptly jumping from rest to a closed grasp, for instance. The state of the prediction hand was set via UDP at approximately 32 Hz.

### Software interface

To facilitate a smooth development experience for future work, we built our software framework in Python. This framework is designed to be flexible and accommodate a wide range of experiments by providing only the necessary components such as a graphical user interface (GUI) template that can be edited and extended, real-time plotting capabilities, and prebuilt connection templates for various Input/Output solutions. Movie S3 shows in real time the workflow necessary for setting up and recording three motor dimensions from the third individual with amputation.

For this work, we subdivided the software interface into three distinct panels: input, output, and algorithm/processing. Each panel could only be displayed one at a time. This design choice was made to ensure that users could focus on fewer steps at a time and minimize human errors during hospital visits.

The first panel was used to connect to the Wi-Fi amplifier and to set the recording configurations (see the “Bracelet” section for details). Once connected, the signals were displayed in real time on the right side of the interface, allowing the user to adjust the bracelet if necessary. Connection to the visual interface via UDP was established in the second panel. The last panel contained all necessary functionality to record training data, train, and validate the myocontrol algorithm. The GUI for recording training data allowed users to select the recording duration and choose the movements they wished to display when executing their chosen movements. The displayed movement could either match the executed one or differ, depending on how well the participant remembered a particular movement. We chose 30 s per movement and selected the movements after discussing with each participant which movements they could remember. Generally, we found that grasping and flexing the index finger were not forgotten by the participants, while flexing the pinky finger individually was often imagined as flexing the ring and pinky fingers together.

The wireless amplifier streamed the EMG signals at 111 Hz and recorded them at 2000 Hz. Therefore, for 1 s of recording, the amplifier streamed 111 matrices of 32 EMG channels with 18 samples per channel (9 ms). However, as 9 ms was not enough temporal information, we created a real-time queue that buffered 20 EMG windows, resulting in a total signal length of approximately 180 ms (360 samples). The 32 EMG channels have been bandpass filtered between 10 and 500 Hz at base by the amplifier.

To suppress artifacts and other undesired noise, the 32 channels were restructured into their physical configuration of 16 rows by 2 columns (Fig. 2E). Given that the bracelet forms a loop, the 16 × 2 EMG matrices were circularly padded row-wise: The last row was added on top of the first row, and the first row was appended below the last row to simulate the physical arrangement. Each column was zero-padded to facilitate the application of a 3 × 3 filter across the entire grid. A 3 × 3 blur filter was used to suppress high-frequency noise

$$\frac{1}{4}\left(\begin{array}{ccc} 0 & 1 & 0\\ 1 & 0.5 & 1\\ 0 & 1 & 0 \end{array}\right)$$ (1)

This filter, at any given electrode position, could only consider the current electrode and its three immediate neighbors. One RMS value was then computed for each channel and saved together with the mean of the control hand state (Fig. 2B). This resulted in 32 features that corresponded to only one 9D hand state vector.

From experience, we knew that reaching the rest state after attempting a movement is difficult for individuals with SCI due to spasticity (*30*), which involves involuntary overactivity of muscles. To address this, we defined the rest state as occurring when the control hand was below the 50% mark of the fully flexed state for any movement. Anything at or above 50% was then classified as movement. The first 80% of the data was allocated for training, with the remaining 20% reserved for testing. Mean and SD were computed from the training data and used to normalize both the testing set and all data used for the real-time prediction. Using this training dataset, we used a new CatBoost model (*21*) trained on an NVIDIA RTX 4090 Laptop graphical processing unit for each participant individually. Training lasted for 1000 epochs, resulting in approximately 5.21 ± 0.12 s (computed with 120 s of EMG data for *n* = 1000 runs) of training time. To enable the rapid exploration of potential controllable movements, MyoGestic allows for the removal, extension, or rerecording of data as needed. For example, if a participant is recorded multiple times, the machine learning model’s training from previous sessions can be appended to future interactions. Because of the variability of injuries among participants, cross-participant meta-learning was not applied.

### Conformal prediction

Surface EMG signals are inherently variable (*8*) because of physiological factors such as motor units rotating between actively participating in movement generation and resting to avoid fatigue (*27*), the amount of subcutaneous fat layer thickness a participant has (*31*), or the amount of cross-talk present. For individuals with SCI, further variability is introduced in the form of spasticity (*30*).

Conformal prediction is a statistical framework that provides a proxy measurement for uncertainty (*32*). By generating prediction intervals or sets that contain the true outcome with a specified probability, known as the confidence level, it offers more insight into the uncertainty of the model. The size of the prediction set can be used to gauge the model’s uncertainty. In this work, a set containing only one prediction was considered certain. We used the conformal prediction approach of regularized adaptive prediction sets. This method avoids the prediction of empty sets while maintaining a regulated set size due to an applied penalty weighting.

To test whether uncertainty awareness would minimize error and improve the predictions for the participants, we created a real-time capable temporal uncertainty solver that we ran in simulated real time after the experiments. Sets containing more than one possible prediction were filtered by considering the last 75 sets. To derive a single prediction, we counted the occurrence of each prediction within the sets involved and determined the most frequent prediction as the output.

### Deep regression neural network

We adapted our previously published deep learning model (*22*, *23*) to enable proportional control of hand motor dimensions instead of classification. The original model was a 3D convolutional neural network (CNN) that used five 64-channel grids. Because the bracelet is a 32-channel grid, we modified the network from a 3D CNN to a 2D CNN, keeping the rest of the architecture unchanged.
