# Supplementary material for: MyoGestic: EMG interfacing framework for decoding multiple spared motor dimensions in individuals with neural lesions
Source: Sci Adv. 2025 Apr 9;11(15):eads9150. doi: 10.1126/sciadv.ads9150 (PMC11980827; doi:10.1126/sciadv.ads9150)
Supplement: Supplementary file 1 — Figs. S1 to S3 Legends for movies S1 to S5 [file sciadv.ads9150_sm.pdf]

Science Advances
AAAS

# Supplementary Materials for

## MyoGestic: EMG interfacing framework for decoding multiple spared motor dimensions in individuals with neural lesions

Raul C. Sîmpetru *et al.*

Corresponding author: Alessandro Del Vecchio, alessandro.del.vecchio@fau.de

*Sci. Adv.* **11**, eads9150 (2025)
DOI: 10.1126/sciadv.ads9150

**The PDF file includes:**

Figs. S1 to S3
Legends for movies S1 to S5

**Other Supplementary Material for this manuscript includes the following:**

Movies S1 to S5

**Fig. S1.**

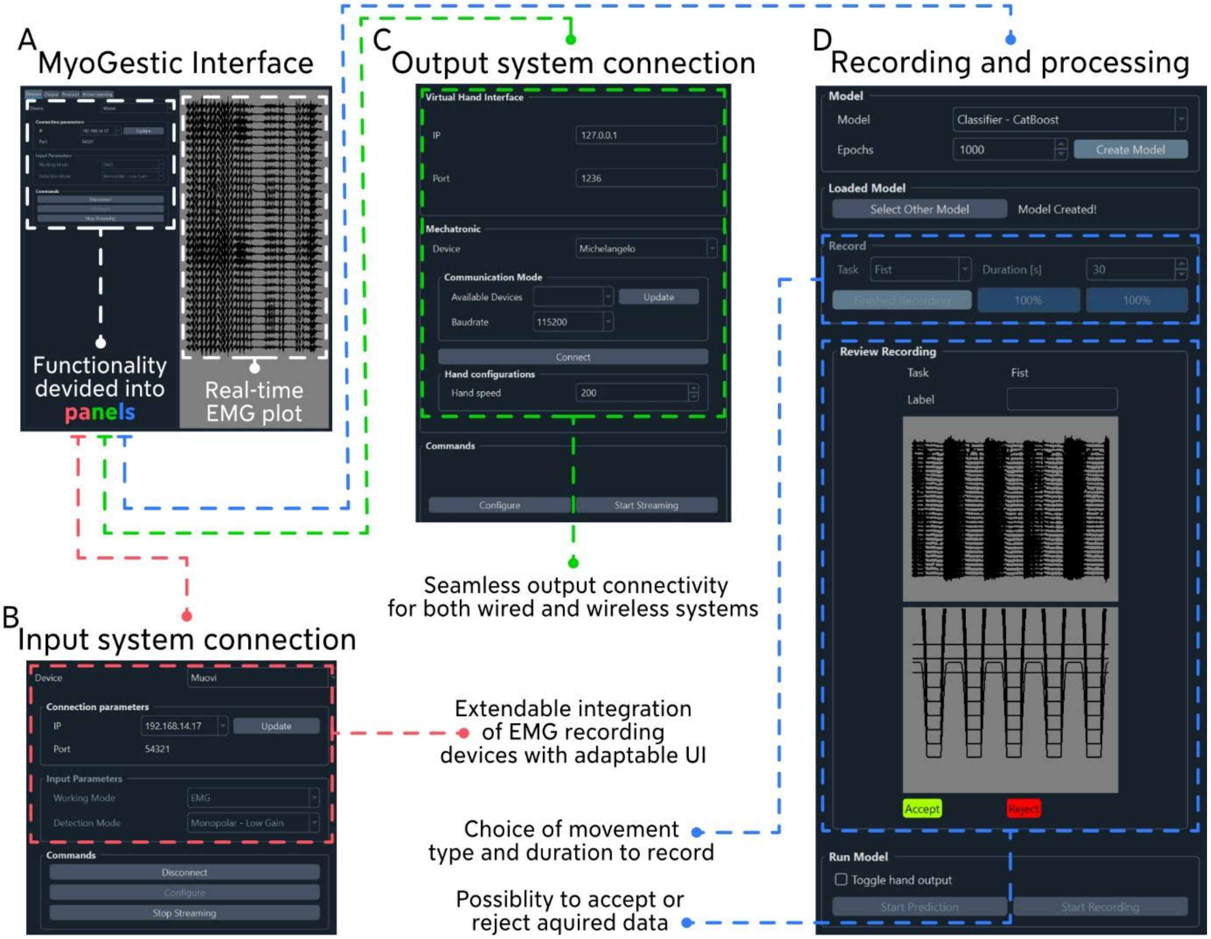


Fig. S1. **MyoGestic interface overview and functionality highlights.** (**A**) The MyoGestic interface streamlines experimentation by guiding both experimenter and participant through each step, providing transparency on current progress and objectives. The functionality (left side) is divided into three main panels: input system (B), output system (C), and recording and processing (D). Each panel can only be displayed one at a time to minimize human error and in turn participant frustration. On the right side, all 32 EMG channels are displayed in real-time, allowing for immediate identification of noise sources to ensure optimal signal quality. (**B**) The first panel connects to the input device, such as our wireless EMG bracelet. The framework is designed to be adaptable and can accommodate any biosignal acquisition device, providing an intuitive solution for integrating the necessary configuration into the user interface (UI). (**C**) The second panel connects to the output device. Just as the first panel any software of hardware output system can be accommodated. (**D**) The final panel manages the experiment workflow, encompassing recording, processing, and validation of myocontrol algorithms. We designed it for ease-of-use, allowing users to record new movements and re-record if necessary (e.g., due to noise or participant errors). This design allows fast accommodation of any participant feedback.

**Fig. S2.**

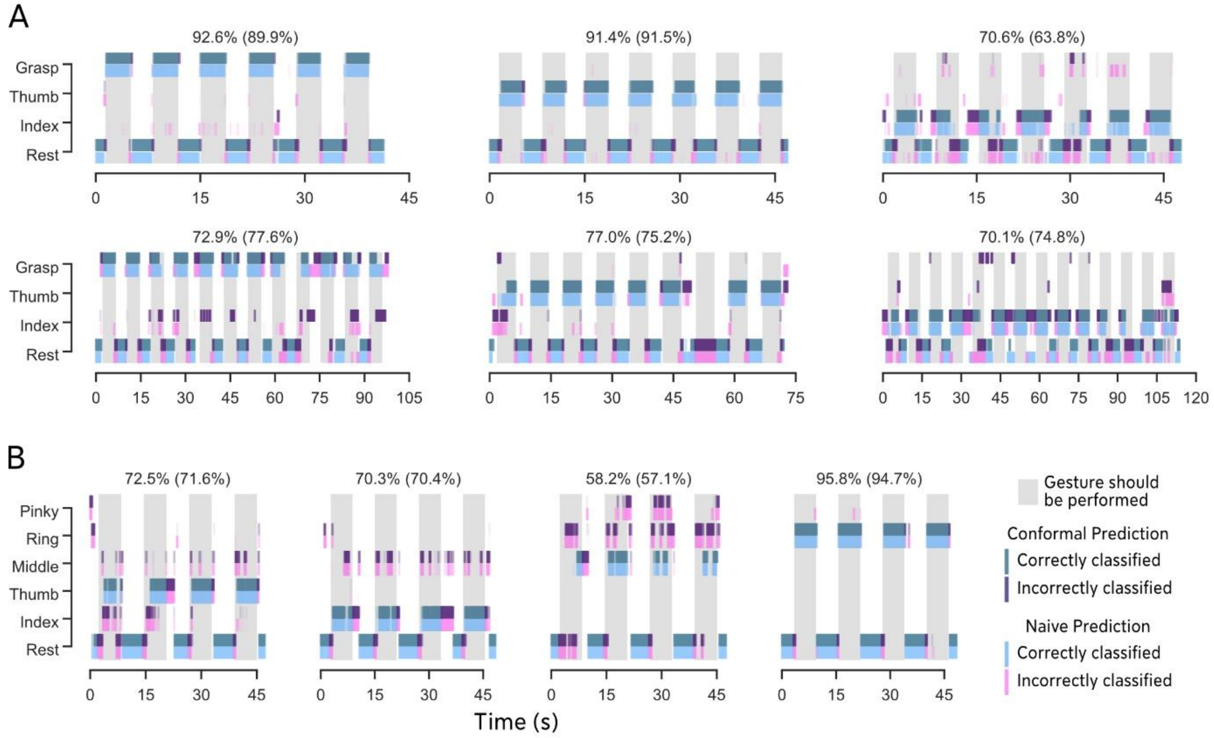


Fig. S2. **Results from the experiments in Fig. 3 & 4 for the other participants not shown.** (**A**) The results for the second and third participants with spinal cord injury (Table 2) from the experiment described in Fig. 3. (**B**) The results for the second transradial amputee (Table 2) from the experiment described in Fig. 4.

**Fig. S3.**

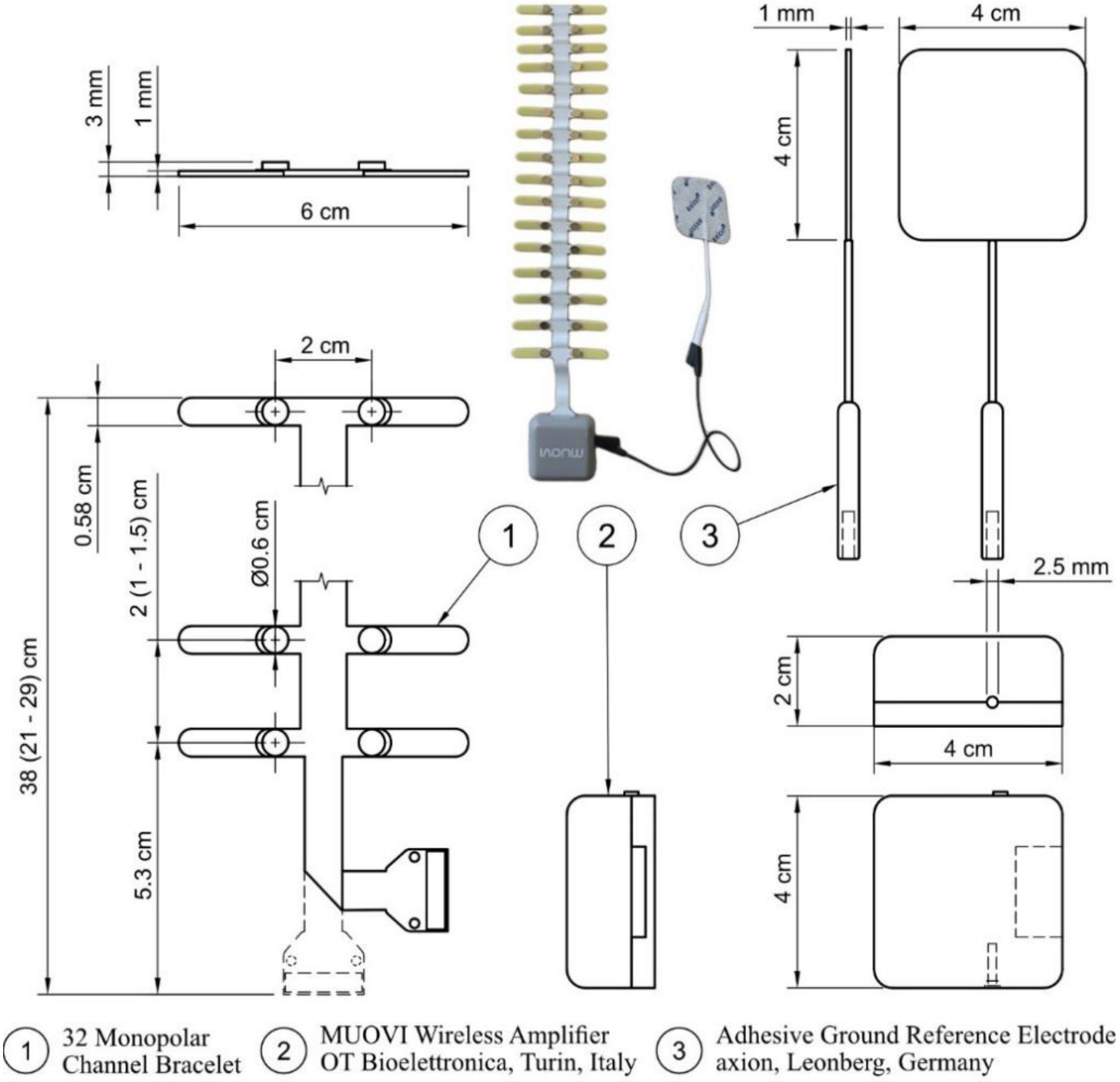


Fig. S3. **Technical schematic of the bracelet components.** The 32 electrodes (①, without fabric sleeve) are arranged in a 16 x 2 configuration affixed on a thin PCB and connected row-wise by a flexible cable. The commercially available Wi-Fi EMG amplifier (②, MUOVI, OT Bioelettronica S.r.l., Turin, Italy) is attached by a custom connector to the electrodes. The amplifier can then be stored in a fabric pocket on the bracelet for ease of wear. The commercially available ground reference (③, Adhesive electrode 4x4 cm, axion GmbH, Leonberg, Germany) is placed on a bone (e.g., elbow) for ideal signal quality and is attached to the amplifier by a 2.5 mm jumper wire.

**Movie S1.**

Real-time full digit control using MyoGestic executed by the participant with a transcarpal amputation. Link: https://youtu.be/NPemwlSg-mE

**Movie S2.**

MyoGestic tutorial shown on an uninjured individual. Link: https://youtu.be/Re3VfgKhjCM

**Movie S3.**

Annotated depiction of the MyoGestic workflow, illustrating the process from software initialization to the control of 3 motor dimensions in a participant with a transcarpal amputation. Link: https://youtu.be/vdP5Ci9cvR4

**Movie S4.**

Real-time control of 4 motor dimensions executed by the third participant with SCI. Link: https://youtu.be/3BvVAu8Nq8c

**Movie S5.**

Real-time control of 5 motor dimensions executed by the first participant with a transradial amputation. Link: https://youtu.be/zxICSVn-3P8
